# Evidence for Phytoremediation and Phytoexcretion of NTO from Industrial Wastewater by Vetiver Grass

**DOI:** 10.3390/molecules26010074

**Published:** 2020-12-26

**Authors:** Abhishek RoyChowdhury, Pallabi Mukherjee, Saumik Panja, Rupali Datta, Christos Christodoulatos, Dibyendu Sarkar

**Affiliations:** 1 Environmental Science and Natural Resources Program, School of Science, Navajo Technical University, Crownpoint, NM 87313, USA; aroychowdhury@navajotech.edu; 2 Department of Civil, Environmental and Ocean Engineering, Stevens Institute of Technology, Hoboken, NJ 07030, USA; pallabi.mkrje@gmail.com (P.M.); spanja1@stevens.edu (S.P.); 3 Department of Biological Sciences, Michigan Technological University, Houghton, MI 49931, USA; rupdatta@mtu.edu; 4 Center for Environmental Systems, Stevens Institute of Technology, Hoboken, NJ 07030, USA; christod@stevens.edu

**Keywords:** insensitive munitions, 3-nitro-1,2,4-triazol-5-one (NTO), industrial wastewater, vetiver grass, phytoremediation, phytoextraction

## Abstract

The use of insensitive munitions such as 3-nitro-1,2,4-triazol-5-one (NTO) is rapidly increasing and is expected to replace conventional munitions in the near future. Various NTO treatment technologies are being developed for the treatment of wastewater from industrial munition facilities. This is the first study to explore the potential phytoremediation of industrial NTO-wastewater using vetiver grass (*Chrysopogon zizanioides* L.). Here, we present evidence that vetiver can effectively remove NTO from wastewater, and also translocated NTO from root to shoot. NTO was phytotoxic and resulted in a loss of plant biomass and chlorophyll. The metabolomic analysis showed significant differences between treated and control samples, with the upregulation of specific pathways such as glycerophosphate metabolism and amino acid metabolism, providing a glimpse into the stress alleviation strategy of vetiver. One of the mechanisms of NTO stress reduction was the excretion of solid crystals. Scanning electron microscopy (SEM), electrospray ionization mass spectrometry (ESI-MS), and Fourier-transform infrared spectroscopy (FTIR) analysis confirmed the presence of NTO crystals in the plant exudates. Further characterization of the exudates is in progress to ascertain the purity of these crystals, and if vetiver could be used for phytomining NTO from industrial wastewater.

## 1. Introduction

Conventional explosives such as 2,4,6-trinitrotoluene (TNT) and 1,3,5-trinitro-1,3,5-triazine (RDX) have been used in weapons for decades. Since the 1990s, however, the focus has shifted to developing formulations of insensitive munitions (IMs), which are safer to handle but remain as effective as conventional explosives [1,2,3]. 3-nitro-1,2,4-triazol-5-one (NTO) is one of the main constituents of IM formulations [4]. It is less sensitive to heat and shock and is safer to handle and transport. Detailed chemical properties of NTO are presented in Supplementary Information (Table S1). NTO is more water-soluble than conventional explosives such as RDX and TNT. The solubility of NTO in water increases from 9.97 to 1989.67 g/L when the temperature increases from 11 to 33 °C [5]. Due to its high solubility, wastewater generated in munition plants containing NTO requires physical, chemical, and/or biological treatment according to regulatory standards before being released into the environment.

As wastewaters produced in industrial munition facilities have the potential to contain residues of explosive compounds and their transformation products, they are subjected to extensive industrial waste treatment processes under regulatory discharge guidelines [6,7,8,9]. These processes can be expensive and inefficient. Aerobic and anaerobic biological treatment processes have been explored for the remediation of NTO in wastewater. Under anaerobic conditions, NTO is biotransformed into ATO (3-amino-1,2,4-triazol-5-one), which requires further treatment based on regulatory standards. In a sequential anaerobic-aerobic biodegradation study, while NTO biotransformed into ATO under anaerobic conditions, ATO later mineralized under aerobic conditions [10,11]. Sorbents such as granular activated carbon (GAC) are ineffective, as NTO carries an electrostatic charge in aqueous solutions and sorbs very poorly to GAC [1,12]. Other processes such as reverse osmosis (RO) and electrochemical degradation for NTO removal either produce concentrated waste streams or additional regulated byproducts [1]. A Fe/Cu bimetal system was used to remove NTO from an aquatic medium, and a pH and a solid-to-liquid ratio-based removal of NTO was reported [4,13]. The phototransformation of NTO in an aqueous medium was also tested [14]. The fate of NTO during biological wastewater treatment was also studied, and the ability of wastewater sludges to promote the biotransformation of NTO to ATO was documented [15].

It is important to develop more effective treatment technologies for wastewater streams containing NTO, since the processes tested so far are expensive, ineffective, or produce harmful byproducts. The objective of this study was to evaluate the potential use of vetiver grass (*Chrysopogon zizanioides* L.) to remove NTO from wastewater. Vetiver is high biomass, fast-growing, perennial grass. It has an extensive root system that can penetrate deeply (3–4 m). Vetiver’s ability to remove various environmental contaminants including various metals and antibiotics is well studied [16,17,18,19,20]. Vetiver was also shown to be effective in the remediation of various explosive compounds from the environment [21,22]. Studies showed that vetiver grass has the potential to remove TNT, RDX, HMX (1,3,5,7-tetranitro-1,3,5,7-tetrazocane), DNAN (2,4-dinitroanisole), and NQ (nitroguanidine) from hydroponic media [23,24,25]. Vetiver’s ability to uptake TNT from the soil in the presence of urea has also been reported [26,27]. Studies showed that plant–microbe interaction plays a significant role in vetiver’s ability to remove TNT from soil [28]. While vetiver has been recognized as an effective candidate for the phytoremediation of several explosive compounds, its potential for removing NTO from water or soil has not yet been tested.

## 2. Results and Discussion

### 2.1. Uptake of NTO by Vetiver Grass

The untreated NTO-wastewater used in this study was alkaline, with a pH of 10.2 ± 0.1 (± standard deviation, SD). NTO concentration in the wastewater was measured as 23,161 ± 135 mg/L (±SD). Nitrate, nitrite, and ammonium-nitrogen concentrations in the wastewater were 1680 ± 185 (±SD), 1.07 ± 0.05 (±SD), and 19.8 ± 2.3 mg/L (±SD), respectively. The wastewater also contained 1.5 ± 0.3 mg/L (±SD) of Na and 44 ± 3 mg/L (±SD) of Ca.

**Figure 1:**
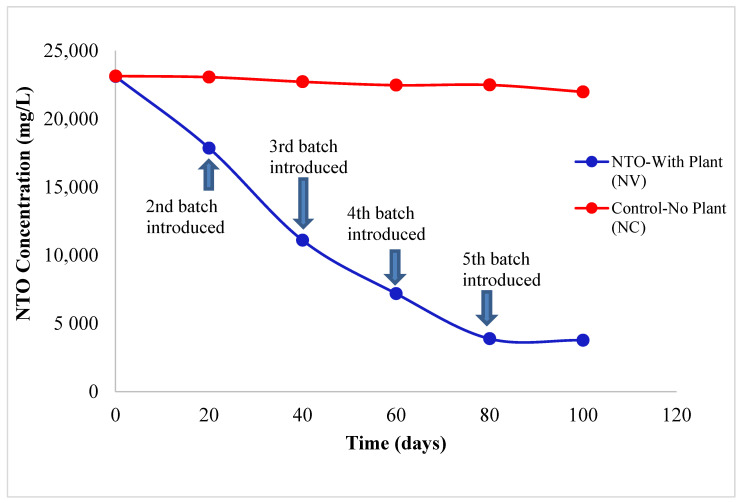
Change in 3-nitro-1,2,4-triazol-5-one (NTO) concentration in NTO-wastewater during the 100 d experiment. New batches of vetiver plants were introduced in NTO with plant (NV) treatments every 20 days. In control experiments (NC), containers of NTO were maintained without vetiver plants. At the end of the 100-day study, the average NTO concentration was reduced by 83.68 ± 0.43% (±SD) of its initial concentration in NV treatments, whereas only 5.0 ± 1.3% (±SD) NTO reduction was estimated in NC treatments.

Two different treatments were used for this study: (1) vetiver grown in NTO-wastewater (NV), and (2) NTO-wastewater without plants (negative control, NC). NV and NC were set up in triplicate, resulting in six containers in total. The duration of the entire experiment was 100 days. Vetiver batches were replaced every 20 days in NV treatment resulting in five successive batch studies. Figure 1 and Table S2 present the trend of average NTO reduction in both NV and NC treatments. At the end of the 100-day study, the average NTO concentration was reduced by 83.68 ± 0.43% (±SD) of its initial concentration in NV treatments whereas only 5.0 ± 1.3% (±SD). NTO reduction was estimated in NC treatments. For NV treatments, the NTO concentration reduced steadily from the first to the fourth successive batches and eventually stayed steady from days 80–100 for the 5th batch of vetiver. Varying amounts of NTO were removed in the NV treatments by successive batches of vetiver. While the first batch removed 22.73 ± 1.07% (±SD) of the initial NTO, later batches removed 51.97 ± 0.56% (±SD), 68.9 ± 0.9% (±SD), 83.22 ± 1.06% (±SD), and 83.68 ± 0.43% (±SD). NTO reduction was measured at the end of the second, third, fourth, and fifth batches, respectively. No significant NTO reduction in NC treatments indicated that NTO reduction was caused by the vetiver plants. Microbiological transformation of NTO to ATO to urea, CO2, and N2 has been reported earlier [2,10,11]. For this study, no transformation products of NTO were detected in the NTO-wastewater in NV treatments, indicating that NTO had been taken up by vetiver over time. Previous studies showed that plants such as big bluestem (*Andropogon gerardii*), Indiangrass (*Sorghastrum nutans*), and switchgrass (*Panicum virgatum*) uptake DNAN and NQ as nitrogen sources and store them in their roots and shoots [29]. RDX bioaccumulates in the edible parts of plants such as lettuce, tomatoes, and corn [30,31,32,33,34,35,36,37,38]. We previously reported that vetiver is capable of taking up TNT from soil and water and degrading it within its tissue [22,26]. A decrease in NTO concentration in NV treatments indicate NTO uptake by vetiver. However, since the NTO-wastewater also contained high levels of nitrate, it is not clear if the vetiver used NTO as a nitrogen source.

The presence of NTO in vetiver roots and shoots was detected in plants collected from NV treatments. It was estimated that on average, vetiver shoots and roots contained 830.9 ± 95.1 (±SD) and 747.8 ± 73.5 mg/kg (±SD) NTO, respectively. The calculated translocation factor (TF) for NTO in vetiver was 1.11, which indicated that vetiver translocated NTO from root to shoot. The presence of NTO inside vetiver roots and shoots indicated that NTO had been taken up by vetiver, and a translocation factor above 1 demonstrates that vetiver translocated NTO from its roots to its shoots. Many studies have previously reported the translocation of explosives such as DNAN, NQ, TNT, and RDX by plants to their above-ground biomass [22,23,27,29,31]. This is the first study, to our knowledge, to report the translocation of NTO in a plant.

**Figure 2:**
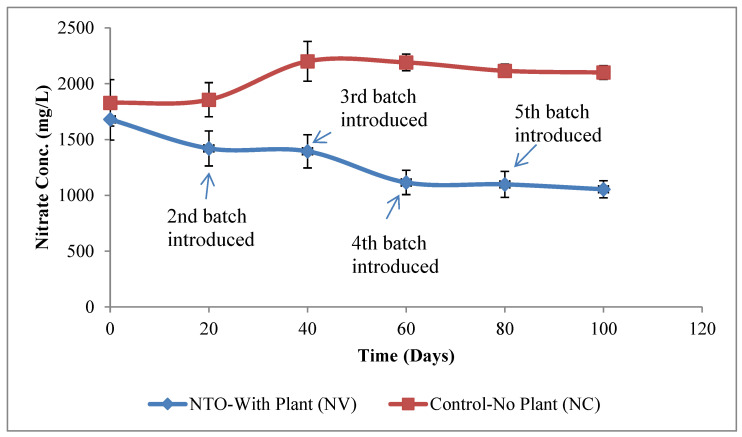
Change in nitrate concentration in NTO-wastewater during the 100 d experiment. New batches of vetiver plants were introduced in NTO with plant (NV) treatments every 20 days. In control experiments (NC), containers of NTO were maintained without vetiver plants. For NV treatments, 37.26 ± 4.52% (±SD) reduction in nitrate in the NTO-wastewater in comparison to its initial value was observed during this study. In contrast, for the NC treatments, nitrate concentration increased by 14.88 ± 3.34% (±SD) on average from its initial concentration during the study.

Figure 2 presents the change in nitrate concentration in NTO-wastewater over time for NV and NC treatments. For NV treatments, 37.26 ± 4.52% (±SD) reduction in nitrate in the NTO-wastewater in comparison to its initial value was observed during this study. Nitrogen is an essential nutrient for plant growth, and the reduction in nitrate in NV treatments is due to its uptake by vetiver plants. In contrast, for the NC treatments, nitrate concentration increased by 14.88 ± 3.34% (±SD) on average from its initial concentration during the study. Nitrate efflux from plants is common and occurs in both stressed and non-stressed plants. Efflux increased in plants that are subjected to mechanical or transplantation stress or changes in the pH of the media [32].

### 2.2. NTO Phytotoxicity Analysis

**Figure 3:**
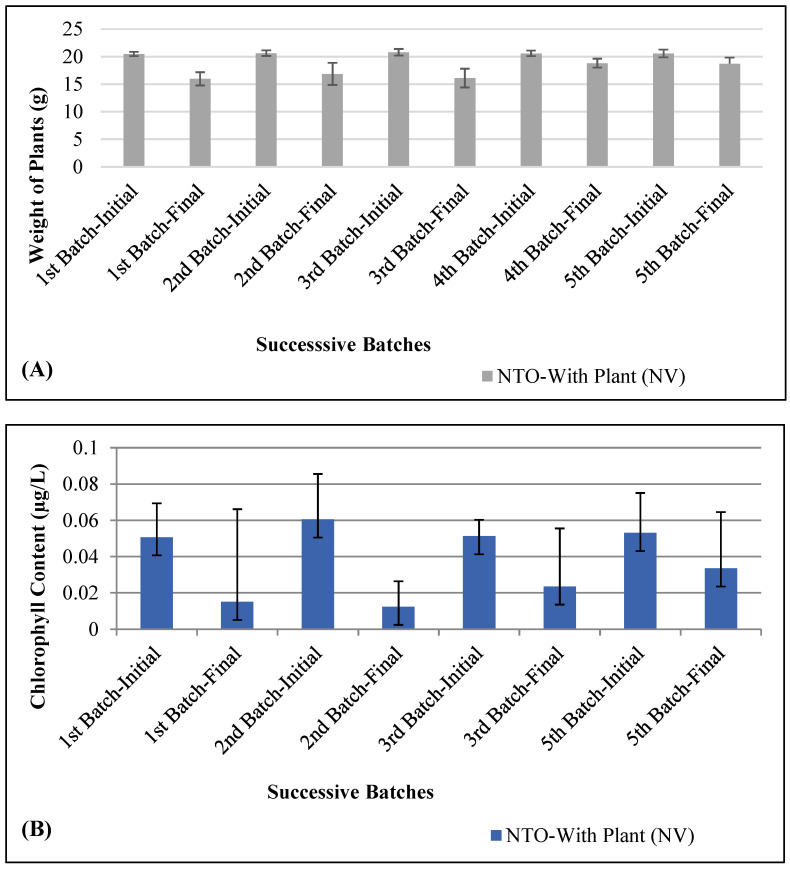
Phytotoxicity of NTO on vetiver grown in NTO-wastewater. (**A**) Plant biomass, (**B**) chlorophyll content. Successive batches of vetiver plants were introduced every 20 days. At the end of each successive batch, on average, vetiver lost 16.07 ± 6.83% (±SD) of its initial biomass in NV treatments. A significant loss in chlorophyll content was observed in vetiver in NV treatments for all batches. An average chlorophyll reduction of 60.18 ± 18.79% (±SD) was noted for the four successive batches.

At the beginning of each successive batch study, vetiver plants were individually weighed before introducing them to the experimental treatment, NV (vetiver grown in NTO-wastewater). Each successive batch of plants introduced was of approximately the same size and weight (21.1 ± 0.7 g (±SD)) as the previous batch. Figure 3A presents the change in plant biomass over time. At the end of each successive batch, on average, vetiver lost 16.07 ± 6.83% (±SD) of its initial biomass in NV treatments. It was observed that in the first three successive batches, plant biomass loss was higher (22.01 ± 0.20% (±SD), 18.2 ± 0.6% (±SD), and 22.5 ± 0.5% (±SD), respectively). However, for the fourth and fifth batches, the loss in biomass was much lower (8.59 ± 0.30% (±SD), and 9.03 ± 0.40% (±SD) respectively). NTO concentration was reduced by 69–83% of its initial value when the last two batches of plants were introduced, which might be within the tolerance range of NTO for vetiver. From the loss in vetiver biomass in the NV samples, it is clear that NTO is toxic to vetiver plants. Our results are similar to other studies on the effect of explosive compounds on plants. Studies showed that the biomass of *L. sativa* was significantly reduced by TNT at a concentration higher than 32 mg/kg [33]. Plant biomass was reduced by 40% and 70% at a TNT concentration of 100 and 1000 mg/kg in comparison to the control, respectively. It was also reported that the growth of *Morella cerifera* was impacted significantly at 30 and 100 mg/L TNT and RDX concentrations [34]. Other studies also reported significant growth inhibition in smooth bromegrass (*Bromus* sp.), switchgrass (*Panicum virgatum*), big bluestem (*Andropogon geraldii*), and blue grama (*Bouteloua gracilis*), due to TNT toxicity [34,35,36,37].

Figure 3B presents the change in chlorophyll content of vetiver during the successive batches for NV treatment (vetiver grown in NTO-wastewater). A significant loss in chlorophyll content was observed in vetiver in NV treatments for all batches. Signs of chlorosis were visible in all the NV treatments. An average chlorophyll reduction of 60.18 ± 18.79% (±SD) was noted for the four successive batches (data for the fourth batch were not analyzed). These results show that NTO is toxic to vetiver and impacts its chlorophyll content. Other studies also reported significant chlorophyll loss in various plants due to TNT and RDX toxicity [33,38].

**Figure 4:**
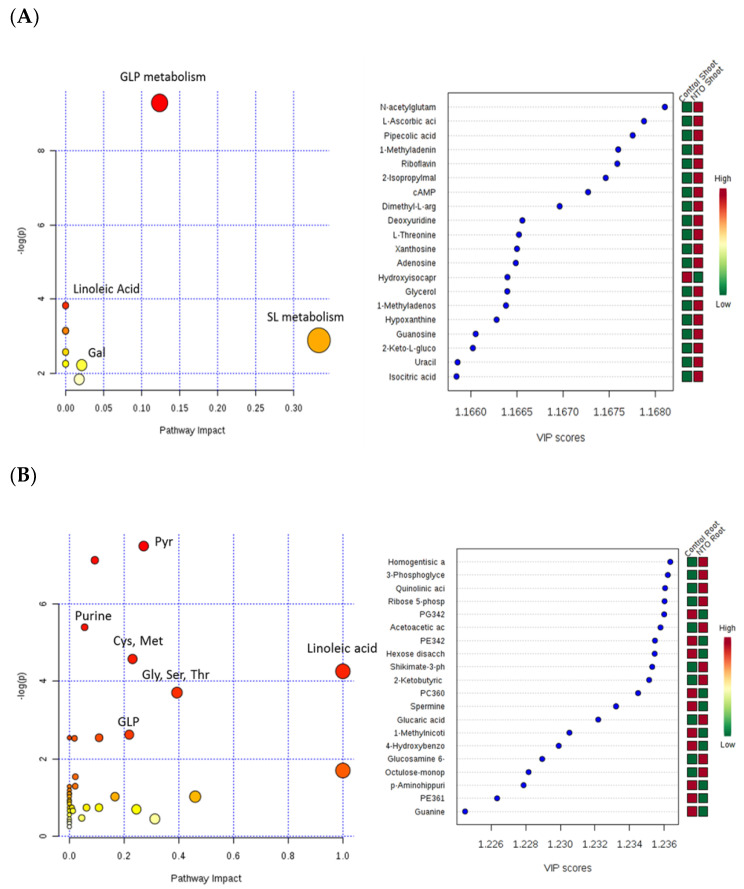
Metabolic profile of vetiver (**A**) shoot and (**B**) root grown in NTO-wastewater treatments (NV) compared to control plants grown in a nutrient medium. GLP metabolism—glycerophospholipid metabolism, Gal—galactose metabolism; Linoleic Acid—linoleic acid metabolism; SL metabolism—sphingolipid metabolism.

The metabolic profiles of vetiver shoot and root tissues exposed to NTO-wastewater (NV treatments) were compared to control tissues of healthy vetiver grown in hydroponic plant growth media (Figure 4). Control tissues showed statistically significant differences in response when compared to treated shoot and root tissue in the PLS-DA model (Figure 4A,B). The major pathways showing upregulation in shoot include (1) glycerophospholipid (GLP) metabolism, (2) galactose metabolism, (3) linoleic acid metabolism, and (4) sphingolipid metabolism (Figure 4A). In the root, the major pathways affected include (1) pyrimidine and purine metabolism, (2) amino acid metabolism (cysteine and methionine) (3) glycerophospholipid metabolism, and (4) linoleic acid metabolism. Figure 4A,B show the significance of the major upregulated metabolites ranked using the variable importance in projection (VIP) score (>1) from the PLS-DA model. The overall metabolic response resembles the osmotic stress response generated by metal or salt stress in plants. Enhancement of galactose and amino acid metabolism could serve to provide osmoprotectants. An increase in lipid peroxidation and membrane damage is indicated by the presence of high levels of phospholipids and linoleic acid. Glycerophospholipids are generated as a result of osmotic stress caused by salt or dehydration [39,40,41]. They act as signaling molecules that trigger several downstream effects that help plants respond to stress. Large increases in the levels of various amino acids have been reported to combat salt and metal stress in vetiver [32,33].

### 2.3. Plant Exudates Analysis

During the five successive batch studies, all NTO-wastewater-treated plants exuded an unknown solid material at the junction of their root and shoot (Figure S1). The amounts of plant exudates varied for the individual batches. The amounts of exudates showed a decreasing trend from the first to the fifth batches. While the highest amount was exuded in the second batch, very little exudation was seen in the fourth and fifth batches. This result indicates that the exudation correlated with the level of NTO in the wastewater. Halophytes excrete salts as well as metals from their salt glands or trichomes on leaves when exposed to high salt or metal-containing media [34,41,42,43]. It was reported that as much as 30%–50% of toxic compounds the plants take up are excreted as a detoxification mechanism to protect sensitive photosynthetic tissue from damage [34,40]. Plant exudates were collected and analyzed by SEM, electrospray ionization mass spectrometry (ESI-MS), and FTIR, and the results were compared with pure NTO to decipher any similarities in structure and composition between them.

### 2.4. Optical Microscopy and Scanning Electron Microscope (SEM) Analysis

**Figure 5:**
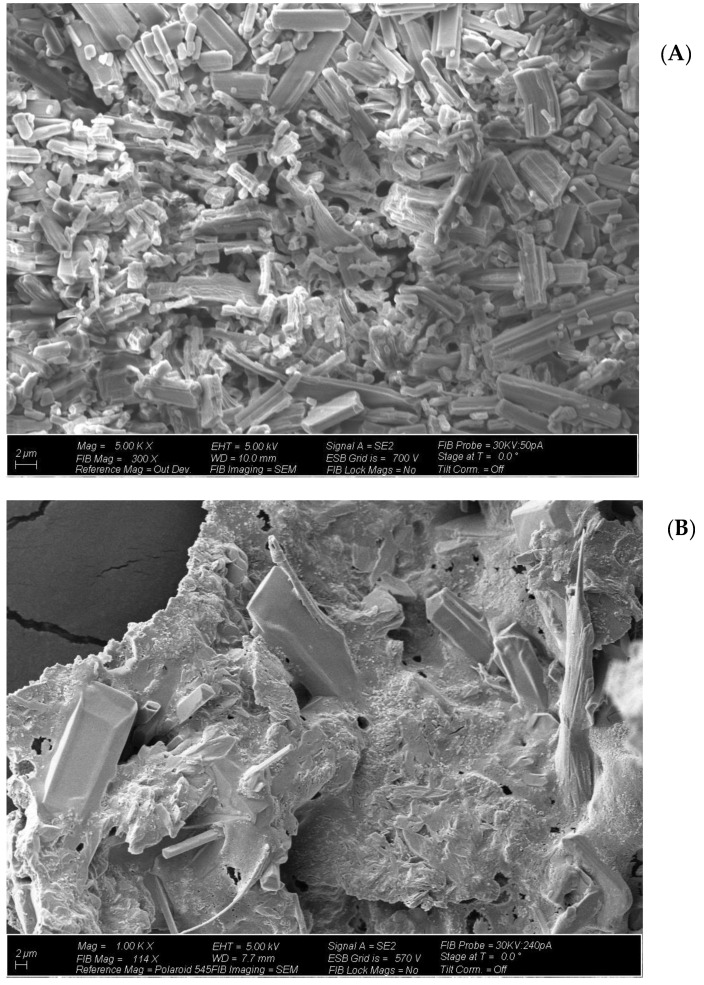
Scanning electron microscope (SEM) image of (**A**) pure NTO solid (5000×) and (**B**) plant exudates (1000×). Distinctive block-like structures were visible when pure NTO particles were examined. The presence of block-like structures was seen in plant exudates, which showed that NTO is a part of the exudates.

Before performing the scanning electron microscope (SEM) imaging, plant exudates were initially inspected under an optical microscope. An AmScope digital microscope imaging camera was used to capture pictures under the optical microscope. Figure S2 presents the optical microscope image of plant exudates. Clear crystalline structures can be seen in this picture. NTO is known to form an agglomeration of rod-like large crystals once exposed to air [44], and our findings are in agreement with the earlier report. Figure 5 represents the SEM image of pure solid NTO. Distinctive block-like structures were visible when NTO particles were examined. To our knowledge, no earlier study has reported an SEM image of pure NTO; hence, it was not possible to compare our result with any other study. Figure 5 also presents the SEM image of plant exudates (dried and ground). It is clear from the picture that the plant exudates were a mixture of many different substances. No specific distinctive structural feature was found under the SEM to identify the composition of this material. The presence of block-like structures was seen under 1000× magnification, which showed that NTO is a part of the exudates.

### 2.5. Electrospray Ionization Mass Spectrometry (ESI-MS) Analysis

**Figure 6:**
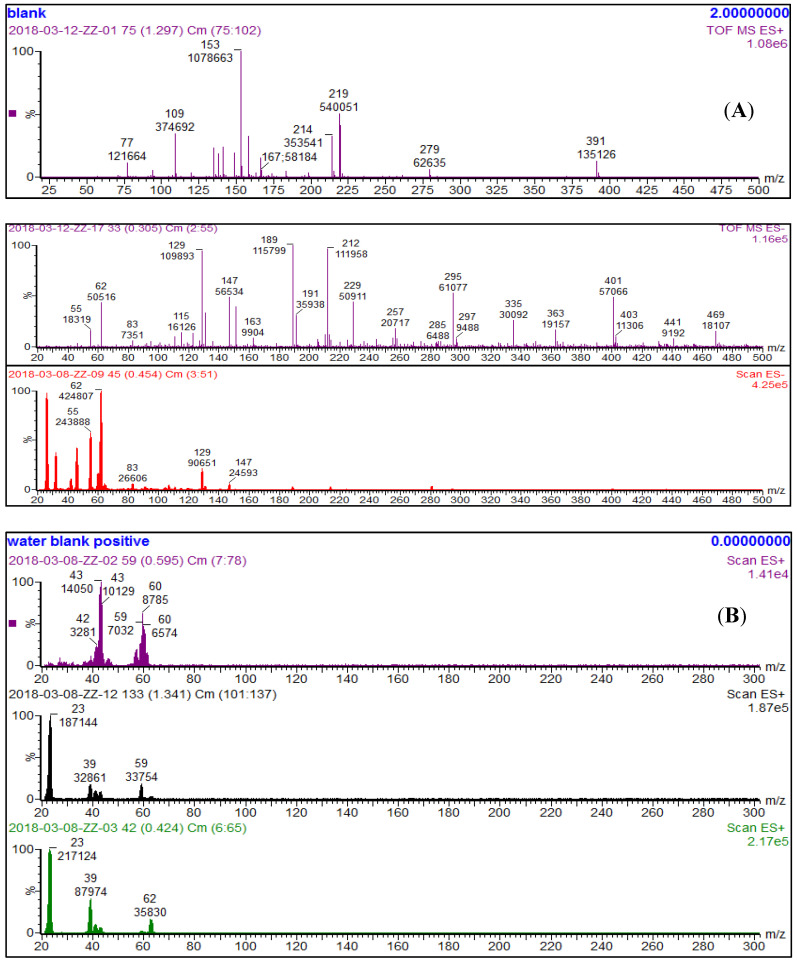
Electrospray ionization mass spectrometry (ESI-MS) analysis of NTO-wastewater and plant exudates at (**A**) negative mode and (**B**) positive mode. Both figures contain a blank (top), NTO-wastewater (middle), and plant exudates (bottom). NTO peak was recorded at an *m/z* ratio of 129 Da in both NTO-wastewater and plant exudates under negative mode.

The ESI-MS analysis of NTO-containing wastewater and exudates was done both in negative and positive mode. The mass to charge ratio (*m*/*z*) was calculated to identify the peaks obtained from the samples. The literature showed that in the negative mode, NTO appears at an *m*/*z* ratio of 129 Da [2]. Figure 6A presents the ESI-MS results of both NTO-wastewater and plant exudates (dissolved in DI water) in the negative mode. The NTO peak was recorded at an *m*/*z* ratio of 129 Da in both NTO-wastewater and plant exudates under negative mode. This result shows that exudates contain NTO particles. As ESI-MS is a qualitative tool, no measurement could be done to quantify the NTO. Several peaks were found in both NTO-wastewater and plant exudate samples. As our NTO-wastewater was an industrial sample, the presence of many other impurities was recorded by ESI-MS spectra. Most of the peaks were found to be adducts of sodium salts (sodium nitrate and sodium carbonate). Figure 6B shows the ESI-MS results of NTO-wastewater and plant exudates in the positive mode. In the positive mode, peaks at m/z ratio of 23 Da and 39 Da position represent sodium and potassium, respectively, which were present in both NTO-wastewater and plant exudates. Our results show that NTO-wastewater contained 1.5 mg/L of total sodium (Na) throughout the study. So, it was clear that sodium, present in the plant exudates, came from the wastewater media.

### 2.6. Fourier-Transform Infrared Spectroscopy (FTIR) Analysis

**Figure 7:**
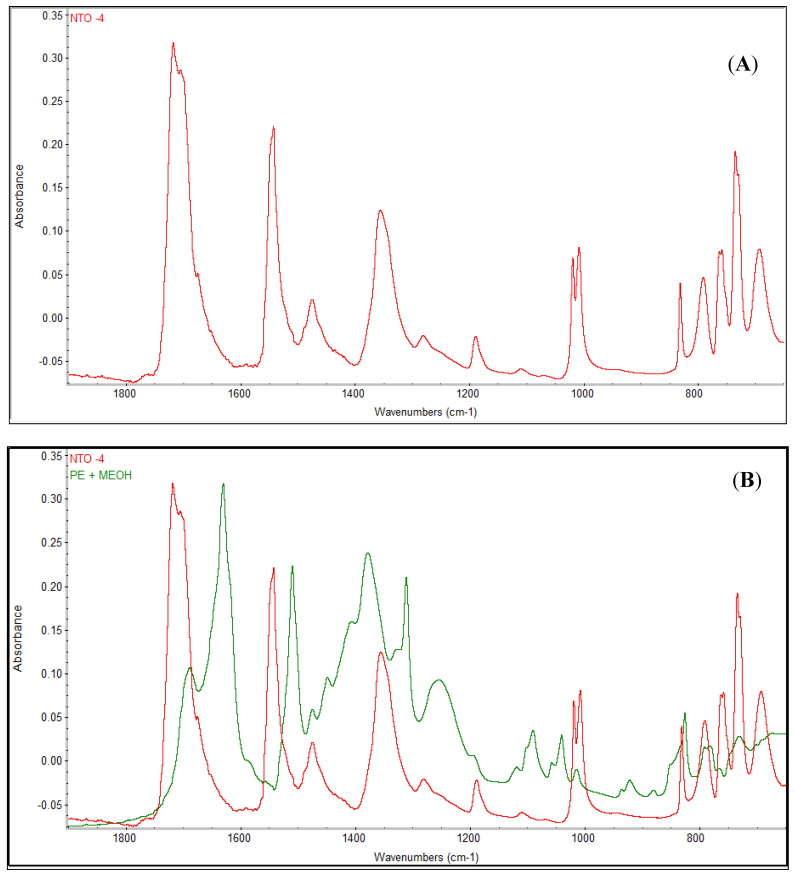
FTIR spectra of (**A**) pure NTO solid, and (**B**) both solid NTO and plant exudates. Peaks at 1800–1600 cm^−1^ (for C=O bonds), 1550–1500 cm^−1^, and 1372–1290 cm^−1^ (for N-O bonds) are distinct for NTO. All three distinct peaks were also visible for plant exudates.

Figure 7A shows the FTIR spectra of pure NTO solids. Based on the chemical structure of NTO, three distinct peaks can be expected from an NTO molecule: 1800–1600 cm^−1^ for C=O, 1550–1500 cm^−1^, and 1372–1290 cm^−1^ for N-O bonds. These three distinct peaks were observed in the pure NTO solids (Figure 7). Figure 7B shows a comparison of FTIR spectra between pure NTO solids and plant exudates. The figure shows these three peaks in plant exudates with a slight shift in position. Several studies have shown that the FTIR peak shift can occur for various reasons, including specific molecular interactions, such as hydrogen bonding, presence of water molecule in the chemical structure, and dipole–dipole interactions [45,46]. Our analysis showed the presence of Na and Ca ions in the plant exudates. The interaction of these co-existing ions with the original NTO molecules could have attributed to the observed peak shift in the FTIR spectra. In addition, vetiver was grown in NTO wastewater and the interaction between NTO molecules and other ions occurred in the hydroponic media, which might have resulted in the introduction of the water molecule(s) in the structure, which might have resulted in the peak shift. FTIR analysis also confirmed similarities in chemical structure between pure NTO and plant exudates, which establishes the presence of NTO in plant exudates.

Salt-tolerant plant species have been reported to detoxify metals in their tissue by phytoexcreting toxic metals through salt glands or trichomes on their leaves [42,43]. Understanding this process would help in ‘mining’ the exuded metals, which would be an added benefit for phytoremediation applications. We report for the first time the extrusion of a munition compound. Further studies are required to find out if the vetiver system could be used to recover and reuse NTO discarded in the waste stream of industrial munition facilities.

## 3. Materials and Methods

### 3.1. Wastewater Characterization

NTO-wastewater and pure NTO solids were obtained from an industrial munition facility in the US. The detailed characterization of NTO-wastewater was performed before the study. The pH of the wastewater was measured using an PC 700 pH meter, Oakton, Vernon Hills, IL, USA. HACH test kits, HACH Company, Loveland, CO, USA were used to measure the total nitrogen (TN), ammonia-nitrogen (N-NH_4_^+^), and total phosphorus (TP) concentrations of the wastewater sample. Nitrate (NO_3_^−^) and nitrite (NO_2_^−^) concentrations of the wastewater were measured using Dionex ion chromatography (IC) with IonPac AS16 (4 mm × 250 mm, Dionex, Thermo Fisher Scientific, Sunnyvale, CA, USA), equipped with a guard column IonPac AG16 (4 mm × 50 mm, Dionex, Thermo Fisher Scientific, Sunnyvale, CA, USA). The total organic carbon (TOC) concentration of the wastewater sampled was measured using a UV-Persulfate TOC Analyzer Phoenix 8000 (Teledyne Tekmar, Mason, OH, USA). In addition, NTO-containing wastewater sample was analyzed for Na, Ca, K, and Mg using an inductively coupled plasma optical emission spectrometry (ICP-OES, 5100 SVDV, Agilent Technologies, Santa Clara, CA, USA). NTO concentration in wastewater samples was measured using a high-performance liquid chromatography (HPLC, Agilent Technologies, Santa Clara, CA, USA, Infinity Series 1260, equipped with a ProStar 410 Auto-sampler and a DAD detector and coupled with a porous graphite column Hypercarb 7 ram, 100 × 4.6 mm). The flow rate of the mobile phase was at 1 mL/min with an isocratic mixture of water: acetonitrile + 0.1% trifluoroacetic acid of 70:30 (*v/v*). The sample injection volume was 35 μL. The analytical wavelengths were 215 nm. Under these conditions, NTO elutes at 4.2 min. A calibration range from 1 to 50 mg/L was used for the analysis and the wastewater samples were diluted as required. Dilution factors were considered while calculating the final amount. A known quality check (QC) standard was inserted after every 10 samples to validate the efficiency of the analytical procedure. All analyses were done in triplicate.

### 3.2. Experimental Setup and Analyses

Vetiver grass (*Chrysopogon zizanioides* L.) was purchased from Agriflora Tropical, Puerto Rico, USA. Plants were initially potted in garden soil and grown there for 30 days. The plants were then placed in a hydroponic system in half-strength Hoagland’s solution for 14 days for acclimatization. After 14 days, the plants were removed from the Hoagland’s solution, dried completely using paper towels, weighed, and used for the experiment. The experiment was conducted in 1 L plastic bottles with a working volume of 500 mL. Two different treatments were used: vetiver in NTO-wastewater (NV) (triplicates), and NTO-wastewater without any vetiver plant (negative control, NC) (triplicates). A 4% plant to solution ratio was maintained for each treatment. All vetiver plants were trimmed from their shoots and roots in such a way that all of them were of approximately the same size and weight. No plant growth nutrients were provided for NV treatments. The 100-day-long experiment was conducted in five successive batch studies of 20 days each. After every 20 days, the old batches of vetiver plants were replaced by new batches of plants. Wastewater samples including all replicates were collected at the same time from each of the bottles periodically. Before collecting the samples, each bottle was mixed thoroughly by swirling so that a homogenous solution can be obtained inside the bottles. Samples were collected by submerging the pipette in the liquid part of the bottles. Samples were analyzed for their NTO concentration, and nitrate concentration. For each measurement (NTO and nitrate concentration), samples were analyzed in triplicates, and analyzed concentrations were compared in Microsoft Excel (version 2007) by calculating mean and standard deviation values.

Vetiver plants collected from NV treatments were also tested for NTO translocation inside the vetiver’s body. Both vetiver roots and shoots were collected (in triplicates) at the end of each successive batch and were analyzed separately to determine NTO concentration in them. A 0.5 g sample (root and shoot) was initially ground to a powder with liquid nitrogen. The powdered tissue was transferred to a tube and 5 mL of acetonitrile was added to each sample. The tubes were kept on a tube rotator for 24 h. Subsequently, the samples were filtered using a 0.45 µm syringe filter and were analyzed for NTO using HPLC. NTO translocation inside the vetiver was measured by calculating the translocation factor (TF) following the standard protocol [29,47]. The analyzed data were compared in Microsoft Excel (version 2007) and mean and standard deviation values were calculated.

### 3.3. Phytotoxicity Analysis

At the end of every successive batch, phytotoxicity analysis was performed on the plant samples (collected from NTO treatment, NV) by conducting a total chlorophyll study and a plant biomass study. Total chlorophyll (as a combination of chlorophyll a and b) extraction from the vetiver samples was performed following standard protocols [30,48], and the absorbance was measured at 645 and 663 nm using a Cytation 3 microplate reader, Biotek Instruments, Winooski, VT, USA. The weight of each plant was recorded before and after each successive batch study. Before weighing the plants, the roots were dried thoroughly with paper towels.

### 3.4. Plant Metabolomics Study

Vetiver samples were frozen in liquid nitrogen and were stored at −80°C until the metabolomics studies were performed. Metabolites were extracted according to a standard protocol [40] with a few modifications adopted by the earlier published literature [48,49]. Ampicillin (0.5 mg/mL) was added as an internal standard before extraction. Methanol: acetonitrile (50:50) with 0.125% formic acid was used as an extraction buffer. LC-MS/MS analysis was performed on the extracted samples using an ABSciex Qtrap 5500 mass spectrophotometer (Sciex, Framingham, MA, USA) equipped with a Turbo V electrospray ionization (ESI) source, a Shimadzu LC-20A system, and a PAL CTC autosampler following a standard protocol [33,49]. A total of 325 metabolites were targeted in multiple reaction monitoring (MRM) mode. Two injections, one for negative mode (ESI−) and one for positive mode (ESI+), were performed. The dwell time was set at 5 ms. Purified standards were used to optimize the compound-specific MS/MS parameters. Peaks were manually reviewed, and the peak area of each metabolite was intergraded through Multiquant v3.0 (Sciex). All data processing was done following standard protocol 3450. MetaboAnalyst 2.0 (http://www.metaboanalyst.ca) was used for all statistical analyses. Partial least-squares discriminant analysis (PLS-DA) was chosen for multivariate analysis. A VIP score >1.5 was considered as significant.

### 3.5. Plant Exudates Analysis

In all the successive batches, it was observed that vetivers grown in NTO-wastewater treatments exuded substances from their shoots, which eventually deposited at the junction of the plant’s roots and shoots (Figure S1). During the first successive batch study, the plants exuded the material starting from the fifth day of the experiment. For different batches, the amount of total exuded material varied. Plants exuded the highest amount of material in the second batch of the study. The rate of exuded material subsequently decreased, and a very small amount was collected during the fourth and fifth batches of the study.

At the end of every successive batch study, plant exudates were carefully scraped off the plant surface and properly stored. The weight of the collected solids from every treatment was measured and noted. Special attention was given during the collection process so that no plant shoot part was scraped off with the exudates.

Plant exudates were completely dried and used for further analysis. Initially, plant exudates were inspected under an optical microscope. An AmScope digital microscope imaging camera (AmScope, Irvine, CA, USA) was used to capture pictures under the optical microscope. As many researchers reported that electron microscopy is a good tool to check the purity and morphology of the energetic compounds, the microscopy scans were performed on both exuded solids and pure NTO solids using a field-emission scanning electron microscope Auriga 40 (ZEISS) (SEM, LEO DSM 982, LEO Electron Microscopy, Thornwood, NY, USA).

Electrospray ionization mass spectrometry (ESI-MS) analysis was also performed on both plant exudates (collected from the first two batches of successive batch studies) and NTO-wastewater (obtained from the industrial facility) using a Micromass Quattro Ultima mass spectrometer (Waters Micromass, Manchester, UK) equipped with an electrospray ion source. Many researchers reported that ESI-MS is a reliable qualitative tool to reflect solution-phase structures [50,51]. The ESI-MS comparison of both plant exudates and NTO-containing wastewater was performed to find similarities in chemical structures between these two samples. All analyses were done in triplicates.

Fourier-transform infrared spectroscopy (FTIR) of both plant exudates and pure NTO solids was performed using a Nicolet iS50 FT-IR (Thermo Scientific, Waltham, MA, USA). Both solids were ground to prepare finer particles using a mortar pestle. As plant exudates were moist initially, they were dried in an oven (60 °C for 2 h) before use for FTIR analysis. All analyses were done in triplicates.

## 4. Conclusions

We evaluated the potential of using vetiver grass to remove NTO from wastewater collected from a munition manufacturing facility. In addition to a high concentration of NTO, the wastewater also contained a high concentration of nitrate. The wastewater was treated with five successive batches of vetiver hydroponically, and the batches were replaced every 20 days. The average NTO concentration decreased by 84% in 100 days. In control tanks without vetiver, the reduction was about 5% during the same period. NTO was detected in root and shoot tissues of vetiver, and high translocation from root to shoot was observed. The vetiver plants showed toxicity symptoms such as a reduction in biomass and a decline in chlorophyll when exposed to NTO. Metabolomic studies indicated an increase in lipid peroxidation, membrane damage, and osmotic stress in vetiver exposed to NTO. During the batch studies, NTO-treated plants produced an exudate at the junction of root and shoot. The amounts of exudates showed a decreasing trend from the first to the fifth batches. While the highest amount was exuded in the second batch, very little exudation was seen in the fourth and fifth batches, as NTO levels declined. SEM, ESI-MS, and FTIR spectroscopic analysis confirmed the presence of NTO crystals in the plant exudates, indicating vetiver exudation of NTO as a mechanism to relieve stress in vetiver. Further studies are needed to understand whether any plant or microorganism- mediated biotransformation or degradation of NTO occurs in vetiver. Further studies are also needed to test the feasibility of this technology in large-scale applications under controlled greenhouse environments. If proven feasible in scaled-up settings, existing NTO wastewater holding tanks can be retrofitted with floating treatment platforms of vetiver. At regular intervals, vetiver biomass can be removed and incinerated under controlled conditions. A significant reduction in the total amount of energetics waste is possible by applying this technology at an expense that is much lower than conventional hazardous waste treatment technologies.

## Data Availability

The data presented in this study are available on request from the corresponding author.

## Supplementary Materials

The following are available online, Table S1: Relevant chemical properties of NTO. Table S2: Change in NTO concentration in NTO-wastewater during the 100-d experiment in vetiver grown in NTO-wastewater. Figure S1: Plant exudates deposited at the junction of vetiver root and shoot. Figure S2: Plant exudates under optical microscope. Photos show the presence of crystalline structures that correspond to NTO.
