# Supplementary material for: Evidence for Phytoremediation and Phytoexcretion of NTO from Industrial Wastewater by Vetiver Grass
Source: Molecules. 2020 Dec 26;26(1):74. doi: 10.3390/molecules26010074 (PMC7796298; doi:10.3390/molecules26010074)
Supplement: Supplementary file 1 [file molecules-26-00074-s001.pdf]

Evidence for phytoremediation and phytoexcretion of NTO from industrial wastewater by vetiver grass

Supplementary Information

Abhishek RoyChowdhury [1], Pallabi Mukherjee [2], Saumik Panja [2], Rupali Datta [3], Christos Christodoulatos [4] and Dibyendu Sarkar [2,*]

[1] Environmental Science and Natural Resources Program, School of Science, Navajo Technical University, Crownpoint, NM 87313 USA; aroychowdhury@navajotech.edu
[2] Department of Civil, Environmental and Ocean Engineering, Stevens Institute of Technology, Hoboken, NJ 07030, USA; pallabi.mkrje@gmail.com (P.M.); spanja1@stevens.edu (S.P.)
[3] Department of Biological Sciences, Michigan Technological University, Houghton, MI 49931, USA; rupdatta@mtu.edu
[4] Center for Environmental Systems, Stevens Institute of Technology, Hoboken, NJ 07030, USA; christod@stevens.edu
* Correspondence: dsarkar@stevens.edu; Tel.: +1-201-2168028

Table S1. Relevant chemical properties of NTO (Kim et al., 1998; Badgujar et al. 2008; Felt et al., 2013)

Property	Value
Molecular weight	130 g/mol
Melting point	273°C (decomposition)
Boiling point	295°C
Solubility in water	9.97 g/L at 11°C
	12.8 g/L in water at 19°C
	16.64 g/L at 25°C
	1,989.67 g/L at 33°C
Log K_{ow} at 25°C	0.37- 1.03
Log K_{oc} at 25°C	2.1- 3.03

Table S2. Change in NTO concentration in NTO-wastewater during the 100-d experiment in vetiver grown in NTO-wastewater (NV), and NTO-wastewater without plants (NC) treatments.

	Introduction-1st batch	Introduction-2nd batch	Introduction-3rd batch	Introduction-4th batch	Introduction-5th batch	
	Day 0	Day 20	Day 40	Day 60	Day 80	Day 100
	---------------------------------NTO Concentration (mg/L)--------------------------------					
NTO-With Plant (NV)	23116.74	17862.83	11103.17	7189.80	3879.70	3772.40
Control-No Plant (NC)	23146.63	23064.69	22723.50	22473.90	22493.70	21985.57
NV Treatment-SD	332.50	248.20	129.30	211.30	245.70	99.40
NC Treatment-SD	409.50	195.80	297.90	301.50	345.70	288.60

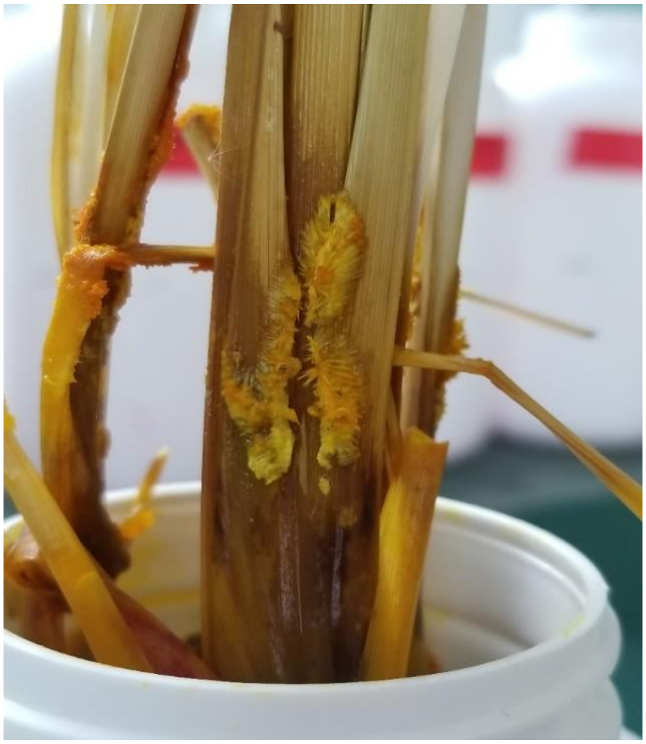

Figure S1. Plant exudates deposited at the junction of vetiver root and shoot.

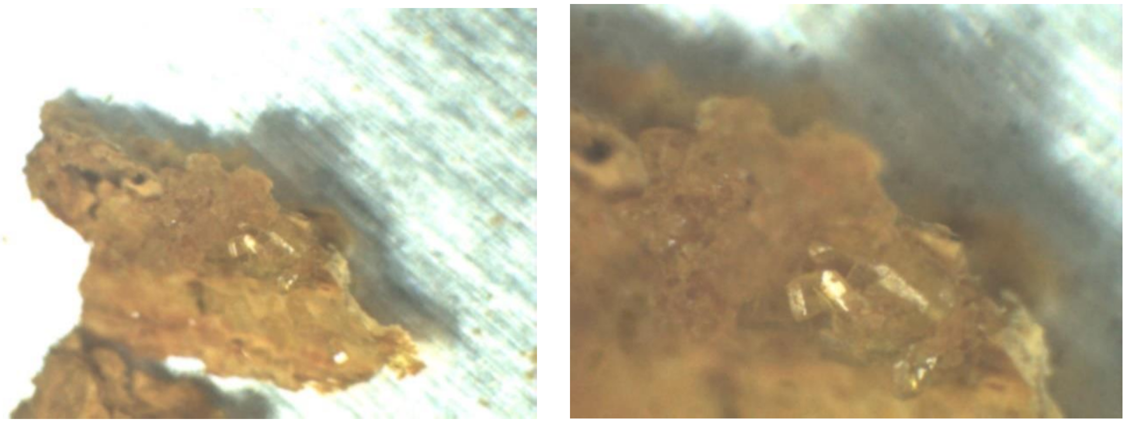

Figure S2. Plant exudates under optical microscope. Photos show the presence of crystalline structures that correspond to NTO.
